# Development of a neutralization assay and bioluminescent imaging mouse model for Dehong virus (DEHV) using a pseudovirus system

**DOI:** 10.1128/spectrum.01557-24

**Published:** 2025-04-02

**Authors:** Xuelian Wu, Fan Liu, Tao Li, Danfeng Li, Yanru Shen, Xiaoai Zhang, Shuo Liu, Qi Jiang, Chenyan Zhao, Jianhui Nie, Youchun Wang, Baomin Feng, Wei Liu, Weijin Huang

**Affiliations:** 1 Division of HIV/AIDS and Sex-transmitted Virus Vaccines, National Institutes for Food and Drug Control (NIFDC) 12540 , Beijing, China; 2 College of Life and Health, Dalian University 74547 , Dalian, China; 3 Guangxi Institute For Drug Control, Guangxi, China; 4 State Key Laboratory of Pathogen and Biosecurity, Beijing Institute of Microbiology and Epidemiology, Beijing, China; 5 Changping Laboratory, Beijing, China; 6 Chinese Academy of Medical Sciences & Peking Union Medical College, Beijing, China; National Microbiology Laboratory, Winnipeg, Manitoba, Canada

**Keywords:** filamentous virus, DEHV, NPC1, psedovirus, neutralizing antibody

## Abstract

Dehong virus (DEHV) is an emerging filamentous virus of considerable interest. However, research involving DEHV remains limited, and no suitable models exist to investigate its pathogenicity or transmission. In this study, we developed an *in vitro* neutralization assay to detect DEHV-neutralizing antibodies, as well as an *in vivo* bioluminescent imaging mouse model based on a pseudovirus system. Our results confirmed that DEHV utilizes the Niemann–Pick disease, type C1 (NPC1) receptor for cellular entry. Additionally, the neutralization assay demonstrated that DEHV antiserum does not exhibit neutralizing activity against Mengla or Marburg viruses. This pseudovirus-based system provides a valuable platform for studying DEHV biology and evaluating therapeutic interventions.

**IMPORTANCE:**

Bats serve as natural reservoirs for diverse filoviruses across Africa, Europe, and East Asia; numerous strains circulate within these populations. Recently, Chinese researchers identified Dehong virus (DEHV), a novel filovirus carried by bats in China. However, the mechanisms underlying the pathogenicity and transmission of DEHV remain poorly understood. Similar to Ebola virus and Marburg virus (MARV), DEHV uses the Niemann–Pick disease, type C1 (NPC1) receptor for host cell invasion. In this study, we utilized a well-established in *vitro* neutralization assay to confirm that DEHV antiserum lacks neutralizing activity against Mengla and MARV pseudoviruses. Furthermore, we developed an innovative in *vivo* bioluminescent imaging mouse model using DEHV pseudovirus, which offers a visually intuitive and efficient platform for evaluating antiviral therapies and vaccine candidates. This model has considerable potential for advancing research into DEHV pathogenesis and treatment strategies.

## INTRODUCTION

The Filoviridae family, commonly referred to as filoviruses, comprises pathogens known to cause acute hemorrhagic fever in primates. This family includes eight genera and 16 species (1); the most notable members are Ebola virus and Marburg virus (MARV), which exhibit high mortality rates ranging from 24% to 90% (2). These viruses represent substantial threats to human health. In addition to Ebola and MARV, new members of the *Filoviridae* family continue to be identified. For example, Bombali virus was detected in free-tailed bats (*Mops condylurus* and *Chaerephon pumila*) in Sierra Leone in 2018 (3). In Yunnan, China, two additional filoviruses, Mengla virus (MLAV) and Dehong virus (DEHV), were discovered in 2019 and 2023, respectively (4, 5). Bats are considered the primary reservoir hosts for these viruses, harboring them while roosting in or near human habitats; this proximity carries potential risks of zoonotic transmission. However, the pathogenicity of these newly discovered viruses in humans remains unclear.

DEHV possesses the largest genome within the *Filoviridae* family (20,934 nt) (4), with genetic characteristics closely resembling those of MARV and MLAV. The DEHV genome encodes seven proteins: an envelope glycoprotein, a nucleocapsid protein, matrix proteins, non- structural proteins, and a viral polymerase. These proteins are organized in the genome as follows, starting from the leader sequence: 3′-nucleocapsid protein (NP), non-structural protein (VP35), matrix protein (VP40), envelope glycoprotein (GP), non-structural protein (VP30), matrix protein (VP24), and viral polymerase (L)−5′. There is partial overlap between VP35 and VP40, as well as between VP30 and VP24. The GP of DEHV is encoded by a single open reading frame and shares 50% sequence similarity with the GP of MARV. Similar to other filoviruses, DEHV utilizes Niemann–Pick disease type C1 (NPC1) as its intracellular receptor (5, 6).

Although DEHV has circulated among fruit bats in orchards for extended periods, no cases of filamentous virus-related diseases in humans have been reported in these areas. This suggests that DEHV pathogenicity in humans is very low (4). However, a comprehensive investigation of DEHV is needed to fully understand its virulence, pathogenicity, and potential health risks, with the aim of assessing its capacity for future cross-species transmission.

In this study, we established a human immunodeficiency virus (HIV)-based pseudovirus system, in which DEHV GP replaces the HIV envelope protein. Using this pseudovirus system, we developed both an *in vitro* neutralization assay and an *in vivo* bioluminescent imaging mouse model. Additionally, we confirmed that DEHV entry into cells requires interaction with the NPC1 receptor. Building on this finding, we assessed the binding affinities between DEHV GP and NPC1 across multiple species and explored potential cross-protection between DEHV and various filoviruses. Our system offers a valuable tool for studies of the mechanisms underlying DEHV infection, as well as its pathogenicity and potential treatments or vaccines.

## RESULTS

**Fig 1:**
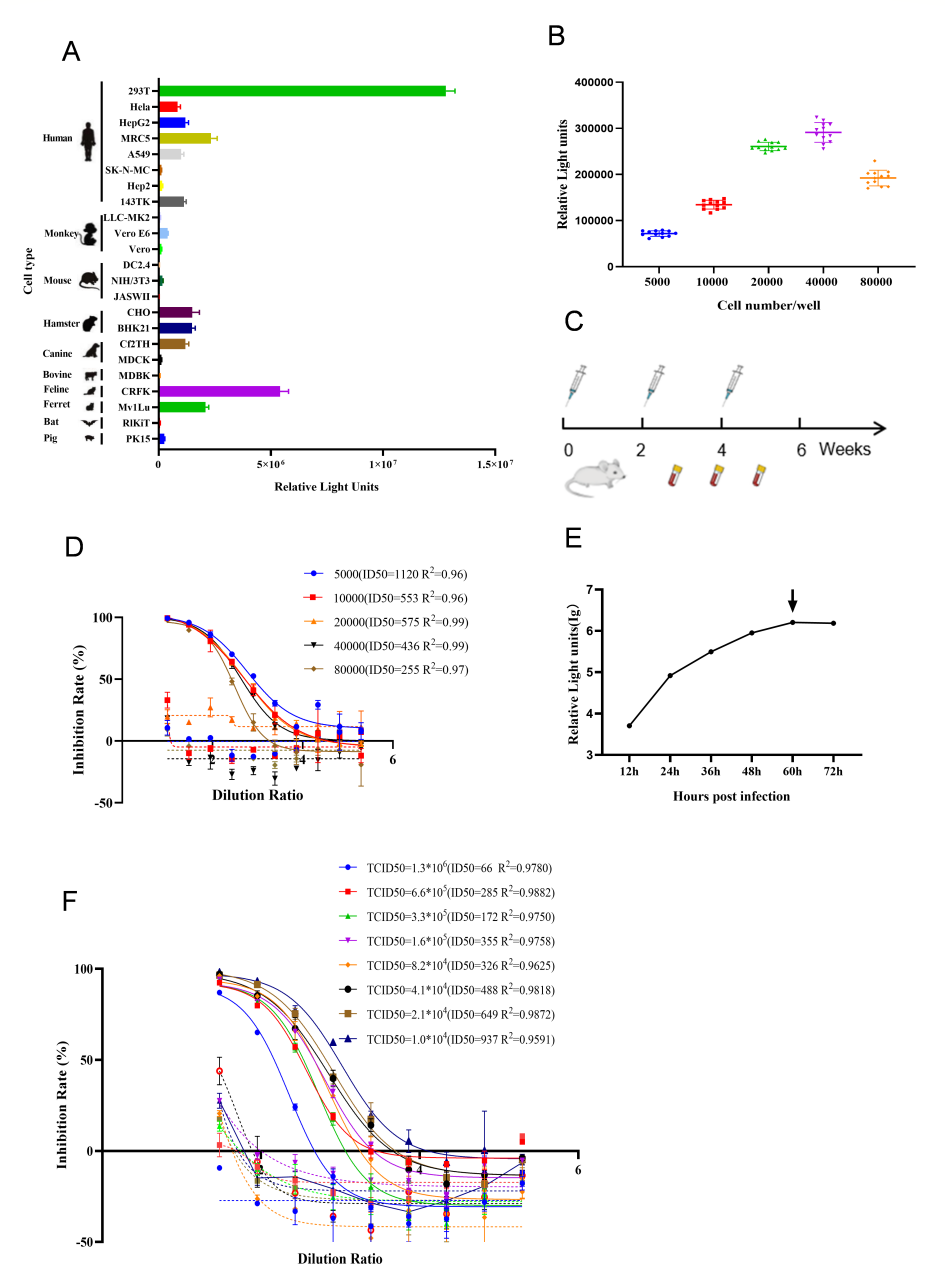
Establishment and optimization of the *in vitro* neutralization assay. (A) Sensitivity of DEHV pseudovirus to different cell types. Cells from various species were seeded in a 96-well plate at 50,000 cells per well. DEHV pseudovirus (40,000 TCID_50_/mL) was added, and bioluminescence was measured 48 h post-infection. (B) Optimization of cell density. HEK293T cells were seeded at densities ranging from 5,000 to 80,000 cells per well in a 96-well plate. DEHV pseudovirus (40,000 TCID_50_/mL) was added, and bioluminescence was measured 48 h post-infection. (C) Diagram outlining the mouse immunization and blood collection process. The first immunization was performed using a DNA plasmid (pcDNA3.1-DEHV-GP vector), followed by two subsequent immunizations with DEHV pseudovirus. (D) Effect of cell density on the sensitivity of the neutralization assay. DEHV pseudovirus (50 µL) was incubated with 7.5 µL of DEHV antiserum for 1 h. HEK293T cells were seeded at densities ranging from 5,000 to 80,000 cells per well, and bioluminescence was measured 48 h post-infection. (E) Optimization of detection time points. DEHV pseudovirus (50 µL) was added to each well of a 96-well plate, followed by the addition of 20,000 HEK293T cells per well. Bioluminescence was measured at various time points post-infection. (F) Optimization of pseudovirus dosage. Mouse serum was serially diluted and incubated with different concentrations of DEHV pseudovirus. The mixture was added to HEK293T cells, and bioluminescence was measured 60 h post-infection. The serum used for this neutralization assay was collected after three immunizations.

DEHV pseudovirus was generated using an HIV-based system (7). Plasmids containing the HIV backbone and the full-length GP of DEHV were co-transfected into HEK293T cells. Cell supernatants were collected 48 h post-transfection to harvest the pseudovirus, and its titer was determined by measuring relative fluorescence values. To evaluate the cell tropism of DEHV pseudovirus, various cell types were infected to identify sensitive cell lines for the detection of DEHV antibodies. The results showed that most cell lines tested supported DEHV pseudovirus infection, indicating broad cell tropism similar to that of the authentic virus (4). Among these, HEK293T cells exhibited the highest luminescence values, suggesting their suitability as a sensitive cell line for neutralizing antibody detection (Fig. 1A). To control for potential variations in luciferase expression due to differences in cell density, we explored the optimal cell-seeding density. The data indicated that densities between 20,000 and 40,000 cells per well produced higher relative fluorescence intensity (Fig. 1B). To generate neutralizing antisera, mice were immunized with DEHV pseudovirus, and serum samples were subsequently collected (Fig. 1C). The specificity of the interactions between the serum and DEHV pseudovirus was confirmed by immunoblotting (Fig. S1A). Further analysis of antibody detection sensitivity revealed that the use of 20,000 cells per well was optimal for neutralization assays. This conclusion was based on evaluations of cell density, 50% inhibitory dilution (ID_50_) values, and determination coefficients (R2) (Fig. 1D). Next, we measured luminescence values at different time intervals while maintaining a constant number of infected cells. The maximum luminescence was observed 60 h post-infection, indicating the optimal time point for neutralization experiments (Fig. 1E). Subsequently, we performed pseudovirus neutralization experiments using varying 50% tissue culture infective dose (TCID_50_) values to determine their impact on antibody detection sensitivity, considering ID_50_ values and determination coefficients (R^2^). A virus dose of 40,000–80,000 TCID_50_/mL was considered optimal for maintaining sensitivity and accuracy in neutralization assays (Fig. 1F). To confirm the specificity of the assay, we conducted neutralization experiments using antisera from mice immunized with Ebola virus and other viruses. The results demonstrated excellent specificity (Fig. S2). These findings confirmed the successful development of a sensitive and specific *in vitro* neutralization assay for DEHV.

**Fig 2:**
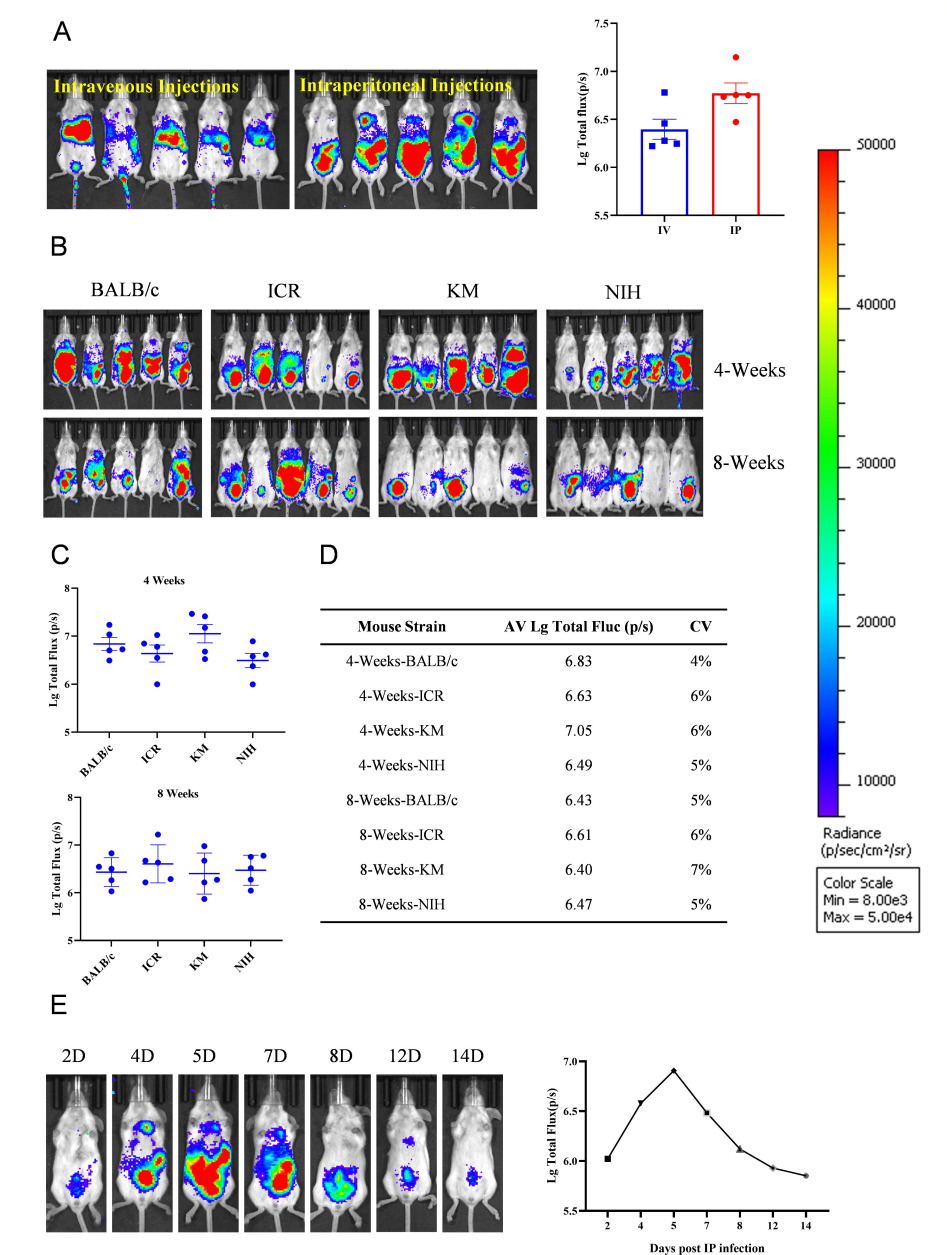
Construction and optimization of the bioluminescent imaging mouse model. (A) Luminescence values in mice injected with DEHV pseudovirus using different methods. The left panel shows imaging results; the right panel presents calculated luminescence values. (B) Comparison of luminescence values among mice of different strains and ages. Mice received intraperitoneal injections of DEHV pseudovirus, and luminescence was measured on the 6th day post-injection. (C) Luminescence values in 4-week-old and 8-week-old mice of different strains after intraperitoneal injection of DEHV pseudovirus. (D) Coefficient of variation (CV) for luminescence values across different mouse groups. (E) Luminescence values detected at various time points after intraperitoneal injection of DEHV pseudovirus, as determined through imaging analysis.

To establish a bioluminescent imaging mouse model for DEHV, we evaluated different viral inoculation methods. Both intraperitoneal injection and intravenous tail injection routes resulted in successful infection of experimental mice. However, intraperitoneal injection was chosen for further analyses due to its stronger bioluminescence signals and procedural simplicity (Fig. 2A). We then assessed the susceptibility of various mouse strains of different ages to DEHV pseudovirus. Based on the high intensity and uniform distribution of bioluminescence signals, 4-week-old BALB/c mice were selected as the experimental model (Fig. 2B through D). Bioluminescent signals became detectable 2 days after infection, peaked on day 5, and declined by day 8 (Fig. 2E). Therefore, the optimal window for analysis was regarded as between days 4 and 7 post-infection. After infection with the pseudovirus, infected and control mice were dissected; their tissues were subjected to pathological analysis. Hematoxylin and eosin staining revealed no differences between pseudovirus-infected and control mice, indicating that DEHV pseudovirus does not cause tissue damage (Fig. S3).

**Fig 3:**
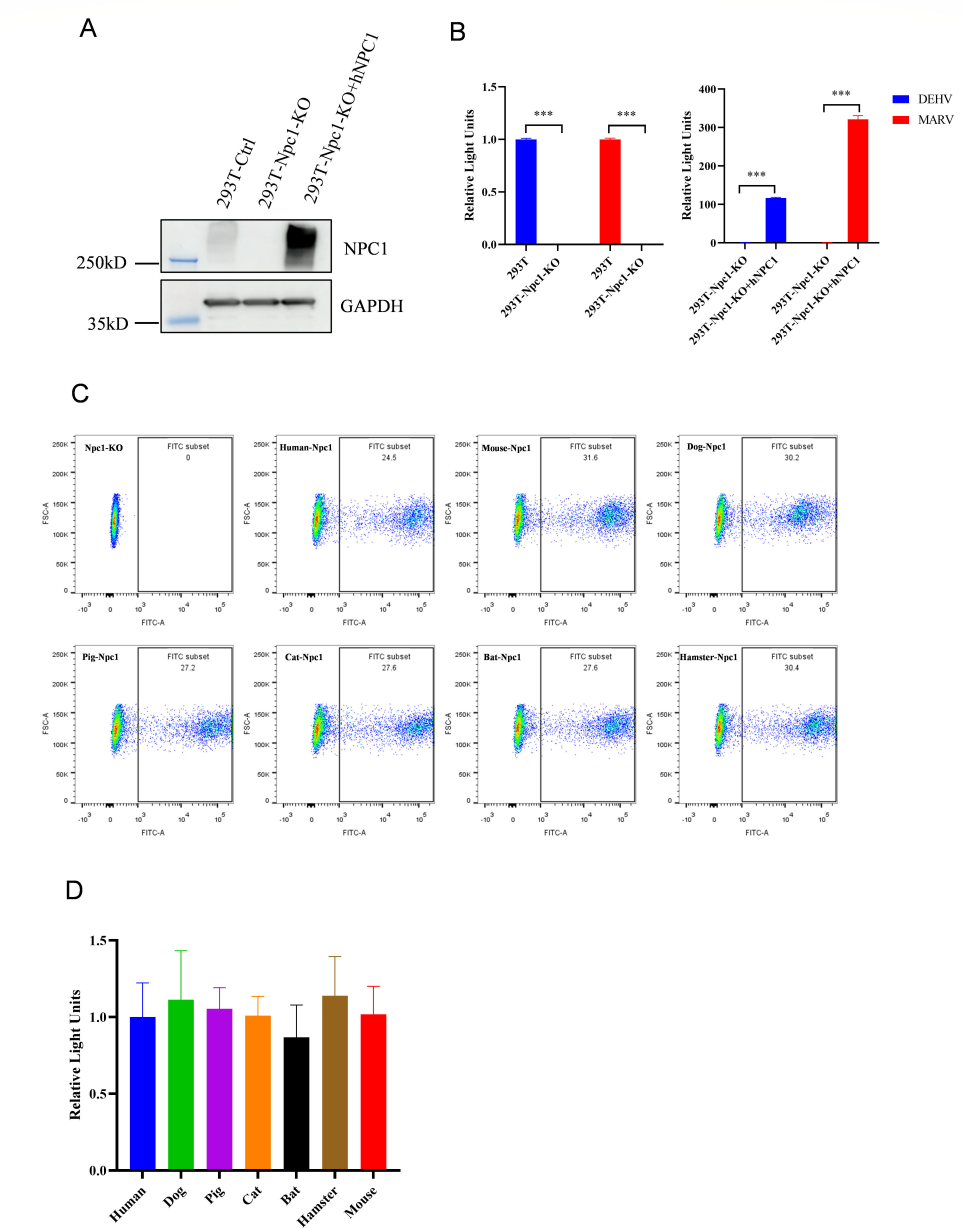
The role of NPC1 in DEHV entry into cells. (A) Western blotting analysis of NPC1 expression levels in different experimental conditions. (B) Infection efficiencies of DEHV pseudovirus in wild-type HEK293T cells, NPC1-knockout HEK293T (HEK293T-NPC1-KO) cells, and HEK293T-NPC1-KO cells transiently expressing human NPC1 (hNPC1). The relative ratios of luciferase values in wild-type HEK293T cells and HEK293T-NPC1-KO cells are shown. (C) Expression of NPC1 from different species in HEK293T-NPC1-KO cells. Cells were transfected with 10 µg of vector encoding NPC1 proteins from various species; GFP expression was analyzed using flow cytometry as a proxy for NPC1 expression. (D) Fluorescence values reflecting DEHV pseudovirus entry into HEK293T-NPC1-KO cells transfected with vectors encoding NPC1 proteins from different species. Data represent four independent biological replicates (*n* = 4).

NPC1, a transmembrane protein located on endosomes and lysosomes (8, 9), has been extensively studied as a receptor for Ebola virus, MARV, MLAV, and other viruses (10–14). NPC1 knockdown reportedly reduces DEHV replication in infected cells (4). To confirm the essential role of NPC1 as the receptor mediating DEHV entry , we generated NPC1-knockout (NPC1-KO) HEK293T cells using Cas9 technology. The knockout was validated through sequencing and Western blotting analysis (Fig. S4; Fig. 3A). DEHV pseudovirus was unable to infect HEK293T-NPC1-KO cells (Fig. 3B), similar to the effect observed with MARV (15). However, overexpression of human-derived NPC1 (hNPC1) protein in NPC1-KO cells restored the abilities of DEHV and MARV pseudoviruses to enter the cells (Fig. 3A and B). These results confirmed that NPC1 functions as the receptor for DEHV entry (4). Notably, the C-terminal domain of NPC1 contains two LOOP regions, which have been previously shown to bind the trimeric GPcl complex of Ebola virus. These LOOP regions are highly conserved across humans and a wide range of non-human species (Fig. S5A) (16). To evaluate the affinity between DEHV and NPC1 proteins from various host species, we constructed plasmids encoding NPC1 from different hosts. The expression levels of eGFP protein indirectly indicate that there are no significant differences in the expression levels of NPC1 across species in HEK293T-NPC1-KO cells (Fig. 3C). Similarly, DEHV pseudovirus utilized NPC1 proteins for cell entry with comparable efficiency across all seven tested species (Fig. 3D).

**Fig 4:**
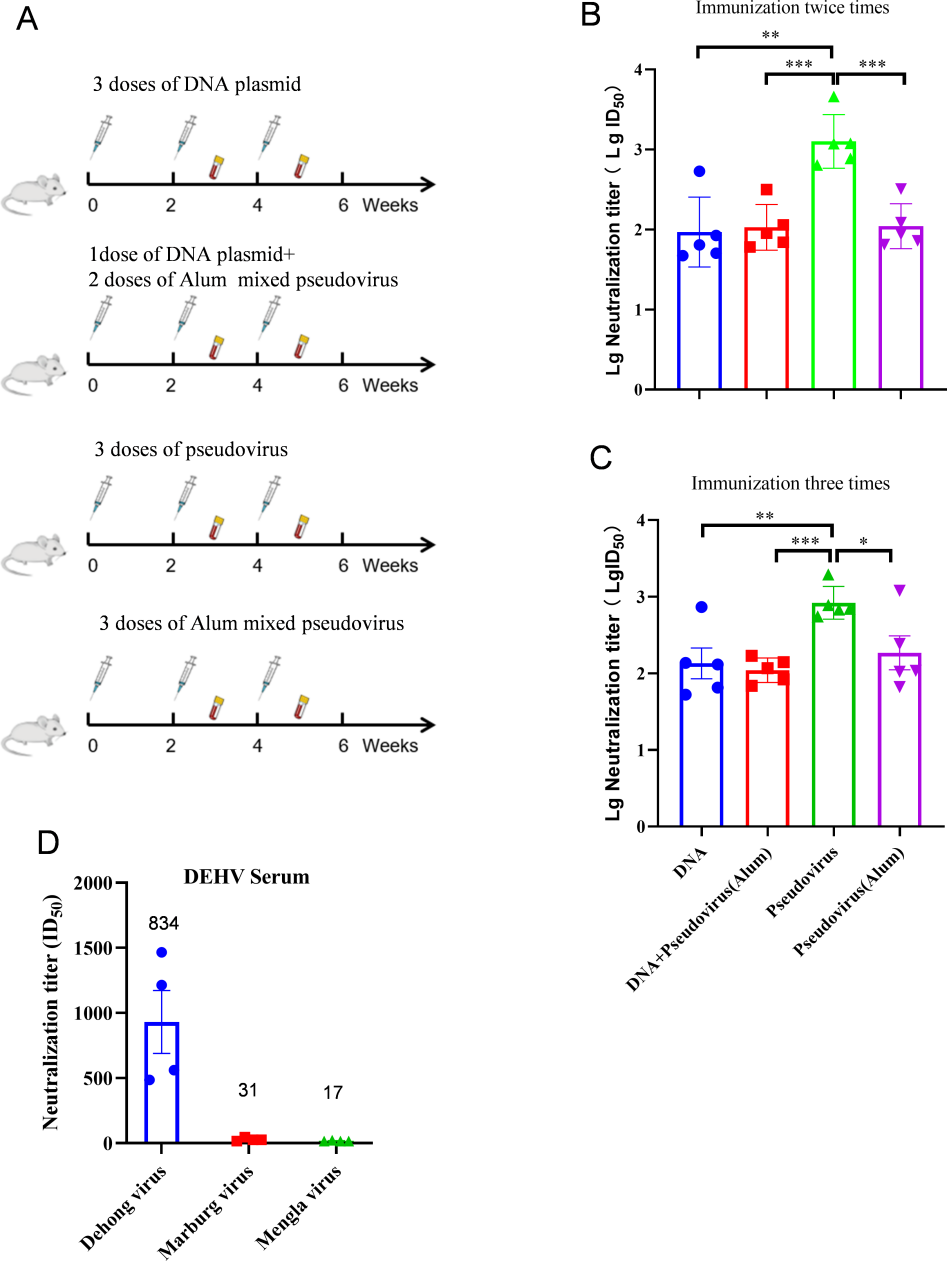
Cross-protection of DEHV antiserum against other filamentous viruses. (A) Diagrams illustrating immunization strategies used in this study. (B) Neutralizing antibody titers in serum after two immunizing doses using various methods (*n* = 5). (C) Neutralizing antibody titers in serum after three immunizing doses using various methods (*n* = 5). (D) Neutralizing antibody titers of DEHV antiserum against DEHV pseudovirus, Marburg pseudovirus, and Mengla pseudovirus (*n* = 4). Geometric mean titer (GMT) values are indicated at the top of each column. In the neutralization experiments, DEHV pseudovirus concentration was 40,000 TCID_50_/mL, Marburg pseudovirus concentration was 400 TCID_50_/mL, and Mengla pseudovirus concentration was 400 TCID_50_/mL.

To generate high titers of DEHV-neutralizing antibodies, we tested various immunization strategies, including DNA vaccine immunization, pseudovirus immunization, a combination of aluminum adjuvant and pseudovirus, and a method involving a single round of DNA immunization, followed by two rounds of aluminum adjuvant and pseudovirus. The efficacy of each method was assessed by measuring neutralizing antibody levels (Fig. 4A). Our assay demostrated that pseudovirus immunization was the most effective method, producing the highest antibody titers in serum samples collected after two rounds of vaccination (Fig. 4B and C). To determine whether DEHV antiserum could cross-protect against other filoviruses, we packaged MARV and MLAV pseudoviruses using the HIV system. Neutralization assays showed that DEHV antiserum exhibited no neutralizing activity against either MARV or MLAV (Fig. 4D).

## DISCUSSION

The Filoviridae family includes several highly pathogenic viruses. In recent years, there has been in increase in the number of filoviruses identified; newly discovered filoviruses include MLAV and DEHV. Although no human infections have been reported, these viruses carry a substantial risk of interspecies transmission due to their broad cell tropism *in vitro*. Despite these risks, gaps remain in our understanding of DEHV, particularly regarding its pathogenicity, virulence, and potential for zoonotic spillover. The development of the DEHV pseudovirus has enabled the creation of a neutralizing antibody assay and an animal model, establishing a platform for future evaluations of antiviral drugs, monoclonal antibodies, and vaccines. Cell receptors play central roles in defining cell tropism and host specificity. In the present study, analyses using the DEHV pseudovirus confirmed that NPC1 is an essential receptor for DEHV invasion, consistent with mechanisms observed in infections caused by the authentic virus.

We also explored the affinities between the DEHV GP and NPC1 proteins from various species using DEHV pseudovirus. Despite mutations in one of the five key amino acid residues within the NPC1 loop of mice, dogs, and hamsters (Fig. S5), no effect on DEHV invasion was observed. This suggests that a single point mutation is insufficient to disrupt binding between NPC1 and DEHV. Additionally, factors that prevent mutations within the C-terminal loop of NPC1 from affecting DEHV invasion activity warrant further investigation in future studies. We observed discrepancies between NPC1 expression levels in different cell types and the efficiency of pseudovirus infection (Fig. 1A; Fig. S6). These differences may arise from variation in the attachment efficiencies of DEHV to surface-binding proteins or factors on different cells. Furthermore, the cleavage efficiency of DEHV-GP across cell types remains unclear and is another topic for future research.

DEHV represents a new species, exhibiting a GP that shares over 50% amino acid identity with MLAV and significant similarity to MARV. However, DEHV antiserum was unable to neutralize MARV or MLAV. Further studies are needed to determine whether MARV antiserum or MLAV antiserum can neutralize DEHV. Intriguingly, our experiments revealed that a three-dose immunization strategy with DEHV pseudovirus produced the highest neutralization titers. The underlying mechanisms of this enhanced immune response require additional investigation.

Our models have certain limitations. The pseudovirus model involves only a single round of replication and contains only the DEHV GP. Consequently, this model is restricted to studies of viral entry mechanisms; it cannot be used to investigate the roles of other viral proteins or replication-related processes. Furthermore, when utilizing small animal bioluminescent imaging models, the potential effects of fur and organ location on imaging results require careful consideration. Despite these limitations, our models provide safe and practical platforms for research concerning prophylactic measures and early interventions involving drugs, antibodies, or non-T-cell vaccines. However, they may not be suitable for studies of T-cell-based vaccines or vaccines that target viruses during replication phases.

In conclusion, we successfully established a pseudovirus-based neutralization assay and a bioluminescent imaging mouse model for DEHV. These user-friendly tools will substantially advance future studies focused on DEHV; they also will facilitate the development of prevention and control measures.

## MATERIALS AND METHODS

### Cells

The following cell lines were used: HEK293T (ATCC, CRL-3216), HeLa (ATCC, CCL-2), HepG2 (ATCC, HB-8065), MRC-5 (ATCC, CCL-171), A549 (ATCC, CCL-185), SK-N-MC (ATCC, HTB-10), Hep-2 (ATCC, CCL-23), 143TK (RRID, CVCL-2270), LLC-MK2 (ATCC, CCL-7), Vero (ATCC, CCL-81), VeroE6 (ATCC, CRL-1586), DC2.4 (Millipore, SCC142), NIH/3T3 (ATCC, CRL-1658), JASWII (ATCC, CRL-3612), CHO (ATCC, CCL-61), BHK-21 (ATCC,CCL-10), Cf2Th (ATCC, CRL-1430), MDCK (ATCC, CCL-34), MBCK (ATCC, CCL-22), CRFK (ATCC, CCL-94), Mv1Lu (ATCC, CCL-64), and PK-15 (ATCC, CCL-33). R1KiT cells (provided by the laboratory of Dr. Zhengli Shi) were cultured in DMEM/F-12 medium with Glutamine (Gibco). HEK293T-NPC1-KO cells were generated using CRISPR-Cas9 technology (OBiO Technology Corp., Ltd.). All cells were cultured in media supplemented with 1% penicillin–streptomycin solution (Gibco) and 10% fetal bovine serum (FBS; TRAN, FS201-02). Cultures were maintained in a humidified atmosphere containing 5% CO_2_ at 37°C.

To create NPC1-overexpressing cells, HEK293T cells were transfected with 10 µg of the pIRES2-EGFP-NPC1 plasmid using Lipofectamine 3000 (Invitrogen), resulting in transiently transfected cell lines (referred to as HEK293T-NPC1).

### Plasmids

NPC1 protein expression plasmids were constructed for eight species: human (Gene ID: 4864), mouse (Gene ID: 18145), dog (Gene ID: 403698), pig (Gene ID: 397591), cat (Gene ID: 493693), fruit bat (Gene ID: 119040988), and hamster (Gene ID: 100689424). The NPC1 sequences from all species were inserted into the pIRES2-EGFP plasmid between the *X*ho*I* and *Bam*HI restriction sites.

The GP genes for DEHV (GenBank: WMZ41126.1), MARV (NC_001608.3), and Mengla virus (NC_055510.1) were cloned into the eukaryotic expression vector pcDNA3.1.

The pSG3.Δenv.cmv.Fluc (envelope-defective HIV-1 SG3 vector) is maintained in our laboratory.

All sequences were codon-optimized for mammalian cell expression, and the protein expression plasmids were synthesized by General Biol. Co., Ltd.

### Quantitative reverse transcription polymerase chain reaction (qRT-PCR)

Previously collected pseudovirus was centrifuged at 4°C and 40,000 rpm for 3 h. The supernatant was discarded, and viral RNA was extracted using the QIAamp Viral RNA Mini Kit (Qiagen, 52906), in accordance with the kit protocol. The RNA was reverse-transcribed into cDNA using the SuperScript III Reverse Transcriptase Kit (Invitrogen, 18080-051). For qRT-PCR, reaction setup and program parameters were configured according to the SYBR Premix Ex Taq Kit instructions (Takara, RR820A). A standard curve was generated from the CT values and the concentrations of pSG3 plasmid to reflect the expression level of GP in the pseudovirus. The PCR primers are listed below.

Primer-F: 5′-TTCGACCGGGACAAAACCAT-3′

Primer-R: 5′-GGGATGATCTGGTTGCCGAA-3′

### Animal experiments

The animal study was approved by the Animal Care and Use Committee at the National Institute for Food and Drug Control (NIFDC; Approval Number: 2023 (B) 055). Four-week-old and 8-week-old female BALB/c, ICR, KM, and NIH mice (Institute for Laboratory Animal Resources, NIFDC) were infected with 1 mL of 2 × 10^6^ TCID_50_/mL DEHV pseudovirus by intraperitoneal or intravenous injection. Bioluminescent signals were monitored at various time points from 2 to 14 days post-infection.

Four-week-old female BALB/c mice were immunized with 50 µg of DNA plasmid (pcDNA3.1-DEHV-GP vector) by electroporation or with 500 pg pseudovirus mixed with 50 µL of aluminum adjuvant by intraperitoneal injection. Immunizations were administered every 2 weeks for a total of three doses. Blood collection began in the third week (after the second immunization), then continued weekly until the seventh week.

### Bioluminescence imaging

Each mouse received an intraperitoneal injection of 100 µL of D-luciferin (100 mg/kg). After 8–10 min, the mice were anesthetized with isoflurane, and luminescence values were detected using the IVIS Lumina Series III Imaging System for small-animal live imaging. Luminescence signals were analyzed using live imaging software to calculate luminescence values. Various tissues were collected from euthanized mice, fixed in paraformaldehyde, and processed for hematoxylin and eosin staining to conduct tissue analysis.

### Pseudotyped virus and neutralization assay

HEK293T cells at approximately 70% confluence were co-transfected with glycoprotein (GP) plasmids from DEHV, MARV, Mengla, or Ebola viruses, along with HIV skeleton plasmids (pcDNA3.1–GP and pSG3.Δenv.cmv.Fluc) at a 1:2 ratio, using Lipofectamine 3000. The medium was replaced 6–8 h after transfection, and cells were incubated for an additional 48 h. Supernatants were collected, centrifuged at 210×*g* for 5 min, filtered, aliquoted, and stored at −80°C for future use.

Pseudovirus titration was performed with an initial threefold dilution in a 96-well culture plate, followed by serial threefold dilutions across six replicates. The final column served as a control containing cells without pseudovirus. Subsequently, 100 µL of HEK293T cells (50,000 cells/mL) were added to each well. After incubation for 48 h in a humidified atmosphere at 37°C with 5% CO_2_, supernatants were aspirated; 100 µL of luciferase substrate (PerkinElmer, 6066769) were added to each well. Plates were incubated at room temperature in the dark for 2 min, then subjected to luminescence measurement using a spectrophotometer (PerkinElmer, Ensight). Relative luminescence unit (RLU) values were used to determine positivity, defined as luminescence at least 10-fold higher than that of the negative control (cells only). The TCID_50_ was calculated using the Reed–Muench method (17).

The outermost wells of a 96-well plate were sealed and left blank. For cell control wells, 150 µL of culture medium was added; 100 µL of medium was added to virus control wells. Serum samples were inactivated at 56°C before use and diluted 30-fold with culture medium in the first row, followed by serial threefold dilutions. Fifty microliters of diluted pseudovirus was added to the sample and virus control wells, and the plate was incubated for 1 h at 37°C. The pseudovirus was prepared to achieve a final concentration of 40,000–80,000 TCID_50_/mL.

Next, 100 µL of HEK293T cells (20,000 cells/mL) was added to each well. After incubation for 60 h, the supernatant was discarded; 100 µL of pre-prepared luciferase substrate (PerkinElmer) was added to each well. The plate was incubated for 2 min at room temperature, then subjected to luminescence signal measurement using a PerkinElmer EnSight spectrophotometer. Data were recorded as RLU values. The 50% inhibitory dilution (ID_50_) was calculated using the Reed Muench method (17, 18).

Inhibition rate = {1– (mean luminescence intensity of sample group – mean cell control) / (mean virus control – mean cell control)} × 100%

Serum log(ID_50_) = log (dilution ratio corresponding to inhibition rate >50%) + (inhibition rate >50% –50%)/(inhibition rate >50% – inhibition rate <50%) ×log (serum dilution ratio).

### Western blotting

Cells were transfected with various plasmids and lysed using RIPA buffer (Cell Signaling Technology). DEHV pseudoviruses were collected and ultrafiltered at 4°C for 20 min using a 30 kDa ultrafiltration tube, then mixed with RIPA buffer for sample preparation. Proteins were separated using 10% sodium dodecyl sulfate–polyacrylamide gel electrophoresis and transferred onto membranes. Samples were incubated with DEHV mouse serum (1:1,000 dilution, followed by an anti-mouse secondary antibody) or NPC1 antibody (Abclonal, A4795, followed by an anti-rabbit secondary antibody). All secondary antibodies were obtained from Beyotime Biotechnology.

### Flow cytometry

Cells transfected with NPC1 from different species were digested, centrifuged, and resuspended in phosphate-buffered saline containing 3% fetal bovine serum. Single-cell suspensions were prepared by filtering the solution through a 40 µm cell filter. Cell counts were performed using the Invitrogen Countess3, and 10^6^ cells were used for flow cytometry analysis (BD FACSAria III). Gating was performed as follows: the first gate identified intact cells, and the second gate selected fluorescein isothiocyanate-positive cells.

### Statistical analysis

Data were analyzed using GraphPad Prism 8.0 software (GraphPad Software). Results are presented as mean ± standard error of the mean. Statistical significance was determined using two-tailed Student’s *t*-tests (***P < 0.001; **P < 0.01).

## SUPPLEMENTAL MATERIAL

Supplemental material spectrum.01557-24-s0001.docx Fig. S1 to S7.

ASM does not own the copyrights to Supplemental Material that may be linked to, or accessed through, an article. The authors have granted ASM a non-exclusive, world-wide license to publish the Supplemental Material files. Please contact the corresponding author directly for reuse.
