# Supplementary material for: Development of a neutralization assay and bioluminescent imaging mouse model for Dehong virus (DEHV) using a pseudovirus system
Source: Microbiol Spectr. 2025 Apr 2;13(5):e01557-24. doi: 10.1128/spectrum.01557-24 (PMC12054171; doi:10.1128/spectrum.01557-24)
Supplement: Supplemental material — Fig. S1 to S7. [file spectrum.01557-24-s0001.docx]

Supplementary

S1: Western blotting of DEHV GP using pseudovirus immunized mouse serum


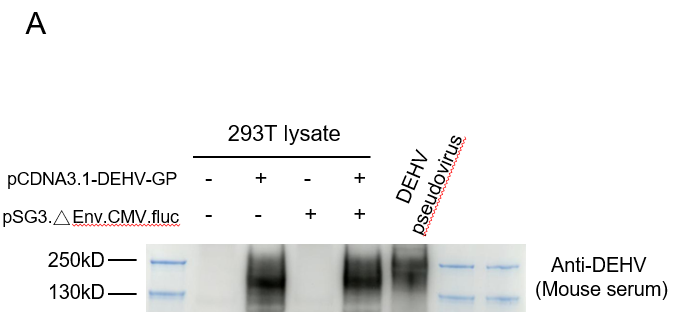


A: DEHV GP was overexpressed in 293T cells by transfection with pCDNA3.1–DEHV-GP, or co-transfection with pCDNA3.1–DEHV-GP and pSG3.△Env.CMV.fluc. Single transfection of pSG3.△Env.CMV.fluc or mock cells with transfection reagents only were included as controls. Sample loading of cell lysate, followed by immunoblotting with mouse serum. Ultrafiltrazione of DEHV pseudovirus followed by Western blotting with mouse serum (Right panel).

S2: Test the specificity of this method using other sera


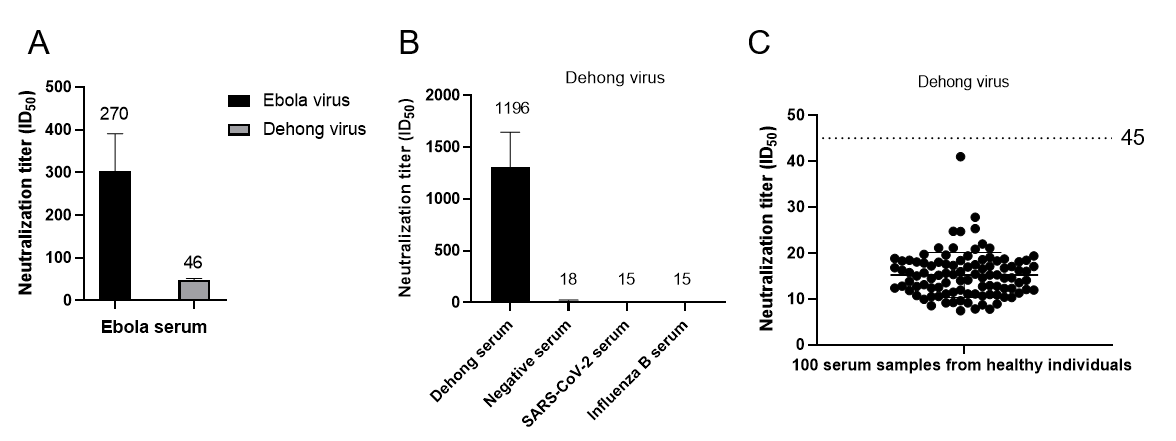


A: Neutralizing antibody titers of Ebola immune serum against Dehong pseudovirus. B: Neutralizing antibody titers against Dehong pseudovirus in immune serum of other species. C: Neutralizing antibody titers against Dehong pseudovirus in 100 healthy human serum samples. The geometric mean titre (GMT) numbers are shown at the top of each column.

S3: H&E tissues slices of various organs in mice injected with DEHV pseudovirus and control


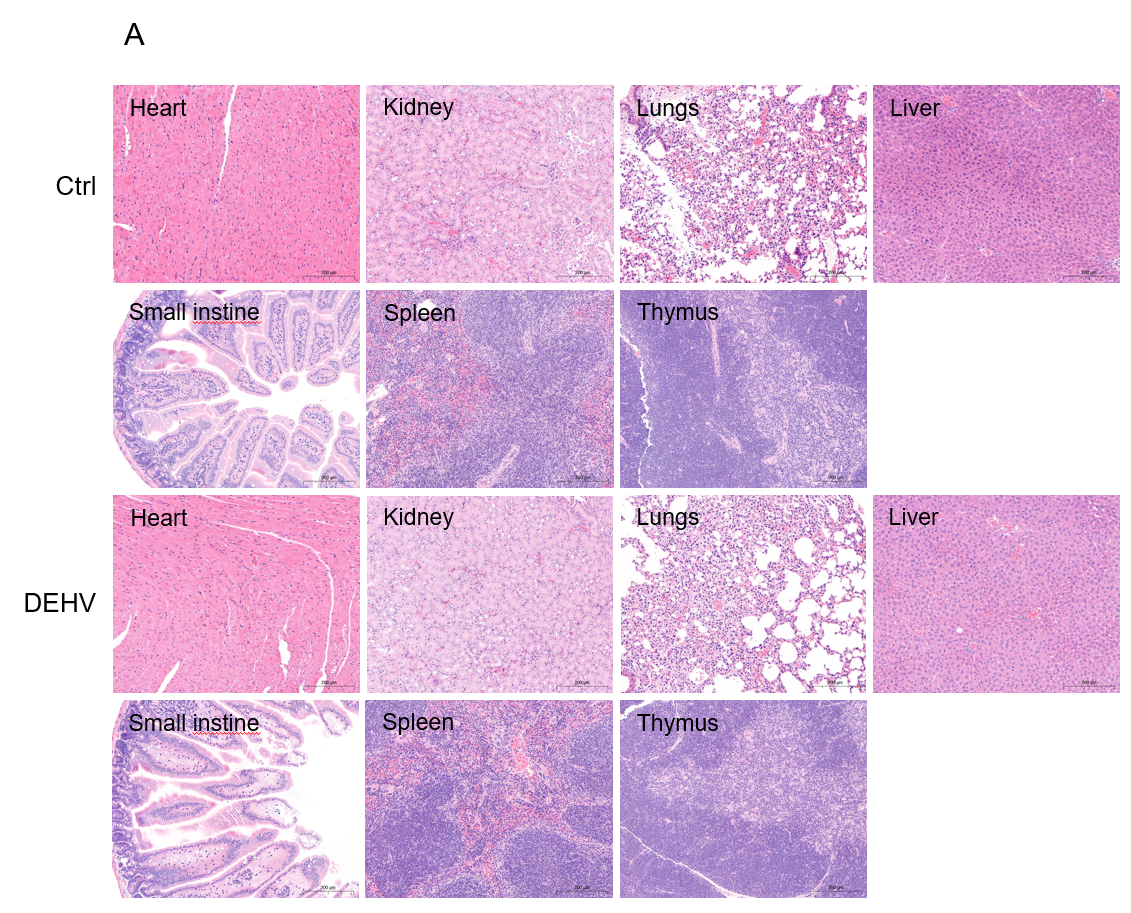


A: After the death of the mouse, the heart, liver, spleen, lungs, kidneys, small intestine, and thymus of the mouse were taken for H&E tissues sectioning and staining.

S4: Sequencing results of NPC1 knockout HEK293T cells


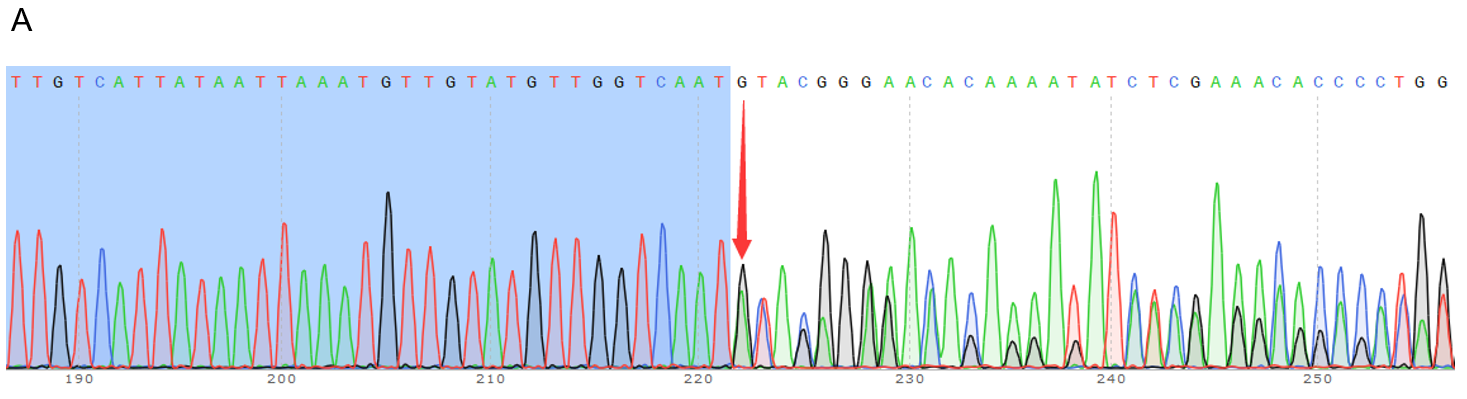


A: The sequencing results show two types of deletions, starting from the red arrow, errors appear in the base sequence, which accurately match with our expectations.

S5: The two LOOP rings in the C-terminal of the NPC1 protein


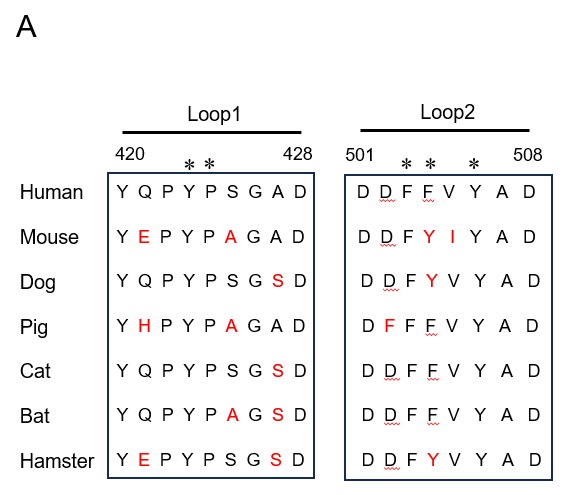


A: Comparison of the core amino acid sequences of the two LOOP rings of NCP1 in humans and animals. Five key residues are marked with asterisks.

S6: The NPC1 expression level in different cell line


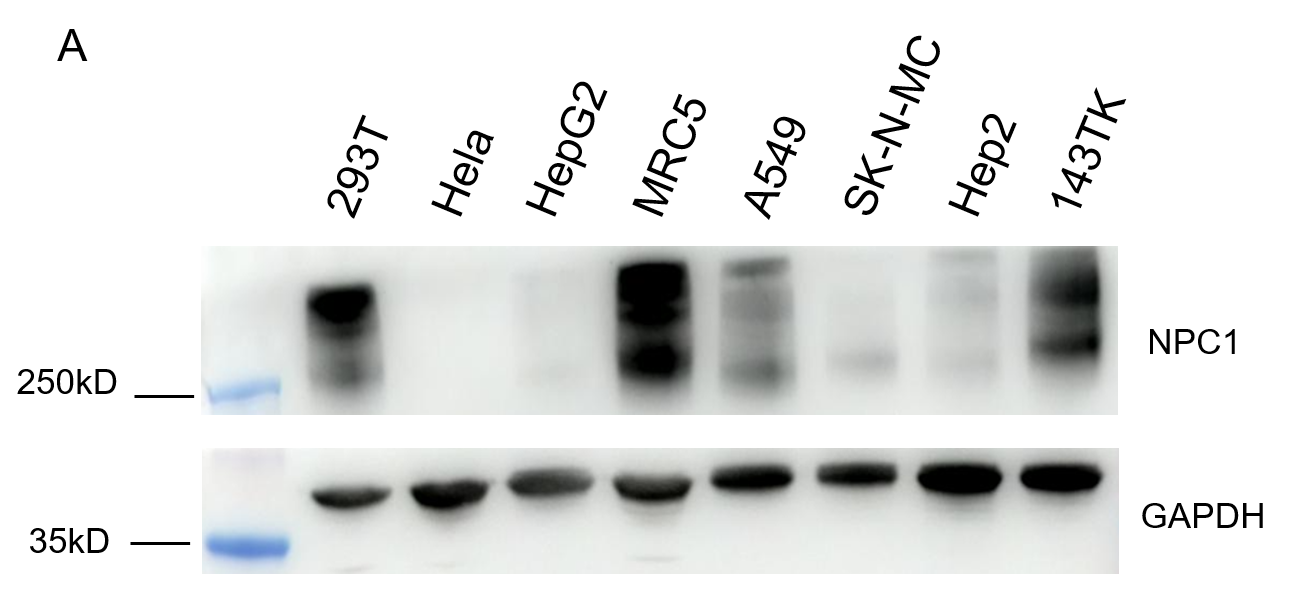


A: The expression level of NPC1 in these cells.

S7 Flow cytometry detection of NPC1 expression levels


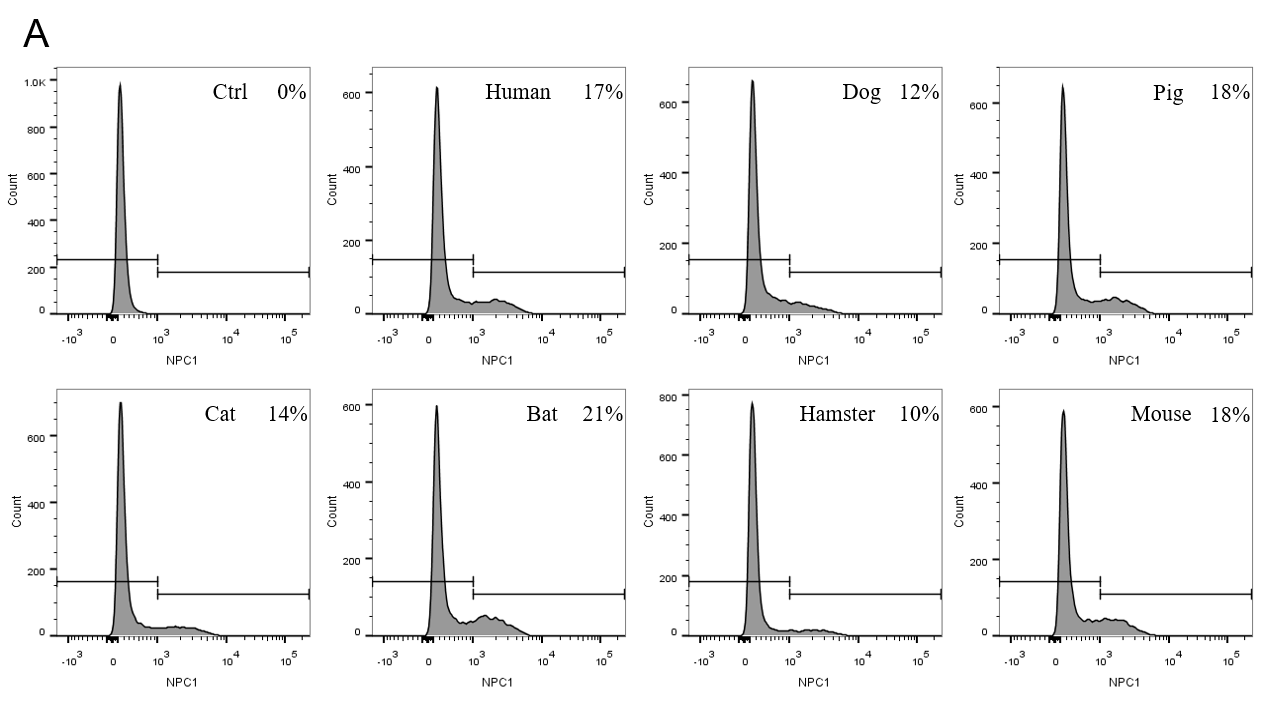


A: Transfect NPC1 plasmids from different species into HEK293T-NPC1-KO cells, then stain with human NPC1 antibody and analyze the expression level of NPC1 using flow cytometry.
